# Evaluation of Microbiome Therapeutic Candidates for Carbapenem-Resistant *Enterobacteriaceae* Associated with Capsule Biosynthesis

**DOI:** 10.4014/jmb.2604.04012

**Published:** 2026-07-30

**Authors:** Ali Atashi, Hoonhee Seo, Hanieh Tajdozian, Faezeh Sarafraz, Md Sarower Hossen Shuvo, Sukyung Kim, Ho-Yeon Song

**Affiliations:** 1 Department of Microbiology and Immunology, School of Medicine, Soonchunhyang University, Cheonan, Chungnam 31151, Republic of Korea; 2 K-Microbiome Institute, 813-26 Yisunsin-daero, 22, Asan, Chungnam 31462, Republic of Korea

**Keywords:** Carbapenem-resistant *Enterobacteriaceae* (CRE), Capsular polysaccharide (CPS) suppression, Microbiome therapeutics, Probiotics, Cell-free supernatants, *Klebsiella pneumoniae*

## Abstract

Carbapenem-resistant *Enterobacteriaceae* (CRE) are life-threatening multidrug-resistant superbugs with severely limited treatment options, highlighting the urgent need for next-generation therapeutics that employ novel mechanisms of action. Microbiome-based therapies have emerged as a promising alternative, with several development pipelines actively progressing. In this study, a comprehensive evaluation of efficacy and safety based on mechanistic evidence is critical for the optimal selection of therapeutic candidate strains. So, we evaluated *Lactobacillus* and *Bacillus* candidate strains for activity against CRE, assessing their efficacy and safety in the context of mechanistic insights. Cell-free supernatants derived from these strains exhibited dose-dependent growth inhibition and time-dependent bactericidal activity against carbapenem-resistant *Klebsiella pneumoniae*. All candidate strains exhibited acceptable safety profiles with no hemolytic activity, although bile salt deconjugation and antibiotic susceptibility patterns varied by strain. Mechanistic analyses revealed that treatment with cell-free supernatants significantly reduced the production of capsular polysaccharide (CPS) and downregulated the CPS biosynthesis-related gene *gal*F, thereby enhancing susceptibility to human serum killing. Additionally, a strong correlation was observed between antibacterial potency and CPS suppression, and two relatively superior candidate strains were prioritized based on their dual functional advantages. Collectively, these findings provide a robust mechanistic rationale for strain prioritization and support the strategic advancement of capsule suppression-based microbiome therapeutics for controlling CRE infections into preclinical and clinical development.

## Introduction

Carbapenem-resistant *Klebsiella pneumoniae* (CRKP) is a leading cause of severe infections in hospital and community settings and is associated with high morbidity and mortality [1, 2]. CRKP is a threat to the health of the entire population as an *Enterobacteriaceae*, particularly in immunocompromised and critically ill individuals [3]. The therapeutic problem is further complicated by the pathogen’s complex virulence arsenal, which not only provides resistance to various classes of antibiotics but also enables the bacteria to evade the host’s innate and adaptive immune responses [3].

Considering the high failure rate of traditional antibiotic-based treatment regimens to eliminate CRKP infections, there is increasing concern about relying on alternative therapy options that function through non-classical mechanisms [4]. In recent years, there has been significant interest in microbiome-based therapeutics, such as probiotics and their bioactive metabolites, as safe and novel methods to combat multidrug-resistant pathogens [5]. These include some of the most widely studied probiotics of the *Lactobacillus* genus, which have been shown to exhibit the widest antimicrobial activity *in vitro* and *in vivo* models [6]. It is noteworthy that certain strains of *Lactobacillus fermentum*, *Lactiplantibacillus plantarum*, and *Lactobacillus sakei* have been shown to prevent a range of clinically relevant pathogens, including CRE, *Staphylococcus aureus*, *Clostridioides difficile*, *Pseudomonas aeruginosa*, and *Streptococcus mutans* [7-9]. Moreover, *Bacillus velezensis* has also demonstrated a protective effect against CRKP infection in murine models, particularly in diabetic population with a high mortality rate [10].

Notably, there is increasing evidence that the antimicrobial effects of probiotics are mediated by secreted bioactive compounds rather than solely by the competition of live bacteria [11]. The secondary metabolites present in probiotic-derived cell-free supernatants (CFS) include organic acids, bacteriocins, peptides, and other small molecules, all of which exhibit potent antimicrobial and antiviral effects [12]. These postbiotic elements offer several translational benefits, including enhanced safety, stability, and controllability, making them desirable as next-generation microbiome-based therapeutics [13].

In this study, we evaluated the efficacy, safety, and underlying mechanisms of cell-free supernatants derived from selected probiotic candidate strains against CRKP. Namely, we examined their antibacterial and bactericidal effects and the mechanism of action by studying their effects on capsule polysaccharide (CPS) and their interference with CPS biosynthesis. This study aims to provide a mechanistic explanation of the rationale for prioritizing strains to be targeted by functional, molecular, and immune-sensitization methods, and to justify the design of microbiome therapeutics based on capsule suppression as a new approach to CRKP infection control.

## Materials and Methods

### Carbapenem-Resistant *Klebsiella pneumoniae* Culture

CRKP was collected from the Pathogenic Resource Bank at Soonchunhyang University Hospital [7]. It was cultured in MacConkey broth (BD Difco, USA) and incubated at 37°C for 18 h. Bacterial growth was determined using a spectrophotometer (DR 1900, HACH, USA), and the optical density at 600 nm (OD_600_) was adjusted to 1.0. Subsequently, the bacterial count was determined using a colony-forming unit (CFU) assay. For future use, a stock culture was prepared in 60% glycerol and stored at -80°C.

### Microbial Culture of Probiotic Strains

*L. plantarum* [14], *L. fermentum* [7], *L. sakei* [8], and *B. velezensis* [10] were obtained from the PMC probiotic bank. Each strain was inoculated into MRS broth (BD Difco) and incubated at 37°C for 24 h in an aerobic shaker-incubator (N-Biotek, Republic of Korea). After incubation, the bacterial density was standardized to an OD_600_ of 1.0 using a Hach spectrophotometer (USA). Species-level identification was confirmed by 16S rRNA gene sequencing prior to further use.

### Preparation of Probiotic Strains Cell-free Supernatant

Probiotic strains were cultured in fresh MRS broth (BD Difco) overnight at 37°C under aerobic conditions and adjusted to an OD_600_ of 1.0. The cultures were then centrifuged at 4,000 rpm for 20 min (Hanil Scientific, Republic of Korea) to pellet the cellular biomass. The clarified supernatants were subsequently passed through 0.22 μm pore-size polyethersulfone syringe filters to ensure the complete removal of viable bacterial cells [15]. The CFS were stored at 4°C for further experiments.

### Determination of MIC of Probiotic Cell-Free Supernatant against CRKP

To evaluate the inhibitory potential of probiotic-derived cell-free supernatants against carbapenem-resistant *K. pneumoniae*, a standardized broth microdilution assay was performed using 96-well plates [16]. Two-fold serial dilutions of the CFS were prepared using fresh MRS broth, and 100 μL of each dilution was dispensed into the wells. The “0” concentration group represented the growth control, consisting of fresh, unfermented MRS broth alone (pH 5.5) without probiotic CFS, rather than a zero-dose dilution of CFS. The CRKP inoculum was prepared from an overnight culture in MacConkey broth (OD_600_ = 1.0), and adjusted to a final concentration of 1 × 10^6^ CFU/mL. A volume of 100 μL of the bacterial suspension was added to each well, and the plates were incubated at 37°C for 18–24 h. Bacterial growth was assessed by measuring the optical density at 570 nm using a Victor Nivo microplate reader (PerkinElmer, USA) [17]. To determine whether the observed antimicrobial activity was attributable to the low pH of the CFS or to heat-labile components, intact CFS was additionally tested in parallel with pH-neutralized CFS (adjusted to pH 7.0 using 10 M NaOH) and heat-treated CFS (100°C for 15 min) under the same concentration range and assay conditions described above. To further confirm that the antimicrobial activity was specific to probiotic-derived metabolites rather than a general property of bacterial CFS, cell-free supernatants from two non-probiotic reference strains, *B. subtilis* and *S. aureus*, were prepared under identical culture and CFS preparation conditions and evaluated in the same assay.

### Time-Kill Kinetics of Probiotic Cell-Free Supernatant against CRKP

The time-kill assay was conducted to assess the antimicrobial effects of the probiotics’ CFS against CRKP. Pure intact CFS, prepared as described before was used in this assay to evaluate the maximal killing kinetics of each probiotic candidate. A CRKP inoculum (300 μL; 1 × 10^8^ CFU/mL) was added to 15 mL of CFS in MRS broth and incubated at 37°C. A control, consisting of CRKP in MRS broth without CFS, served as a growth baseline. At 0, 0.25, 0.5, 0.75, 1, 1.25, 1.5, 1.75, and 2 h, followed by 4, 6, and 8 h, 1 mL aliquots were collected, serially diluted in phosphate-buffered saline (10-fold serial dilutions from 10^0^ to 10^−8^), and plated on MacConkey agar (40 μL of each dilution spotted per plate) to enumerate viable bacterial cells [18-20]. Colonies were counted after overnight incubation, and the results were presented as mean log CFU/mL. The test was performed in triplicate.

### Safety Assessment of Probiotic Strains

The safety and functional properties of the selected probiotic candidates were assessed using hemolysis, bile salt deconjugation, and antibiotic susceptibility assays. To evaluate potential hemolytic activity, fresh probiotic isolates were streaked onto blood agar plates containing 5–10% defibrinated sheep blood (Kisan Bio, Republic of Korea) and incubated aerobically at 37°C for 24 h. After incubation, the type and presence of hemolytic activity were determined through visual inspection [21]. To assess the bile salt deconjugation capacity of probiotic strains, a plate assay was conducted using Taurodeoxycholic acid sodium salt (TDCA; Millipore, Germany) incorporated into de Man, Rogosa, and Sharpe (MRS) agar (BD Difco) at 1 mM. Freshly cultured probiotic isolates were streaked onto TDCA-MRS agar plates and incubated aerobically at 37°C for 5 days. *L. rhamnosus* KCTC5033 and *L. plantarum* KCTC3105 (KCTC, Republic of Korea) served as negative and positive controls, respectively [22]. To determine the antimicrobial susceptibility profiles of the probiotic strains, an E-test (Liofilchem, Italy) was performed on MRS agar. Bacterial lawns were prepared by streaking overnight cultures onto the agar surface, followed by the placement of E-test strips. The plates were then incubated aerobically at 37°C for 48 h [23]. The minimum inhibitory concentration (MIC) for each antimicrobial agent was determined by identifying the point of intersection between the inhibition ellipse and the quantitative scale on the strip. MIC values were interpreted according to EFSA (European Food Safety Authority) cut-off criteria to assess susceptibility or resistance [24]. All tests were conducted in accordance with the South Korea Food and Drug Administration guidelines for probiotic safety evaluation [25].

### Quantification of CRKP CPS Production

Capsular polysaccharide production was measured using the phenol-sulfuric acid method [26]. A co-culture was established by inoculating 10 μL of an overnight CRKP culture into 1 mL of probiotic CFS. MRS broth was served as the experimental control. After incubating for 24 h at 37°C, the cultures were heat-killed at 100°C for 30 min and then centrifuged at 4,000 rpm for 20 min to remove cell debris. The resulting supernatants were mixed with 3 mL of chilled pure ethanol and incubated overnight at -20°C for CPS precipitation. Pellets were collected through centrifugation, re-dissolved in 1 mL of PBS, and treated with 1 mL of 5% (w/v) phenol solution followed by the rapid addition of 5 mL of concentrated sulfuric acid. After 30 min at room temperature, the absorbance was measured at 490 nm. A glucose standard (0.25–1 mg/mL) was used to calculate the CPS concentration. The test was performed in triplicate.

### Assessment of CRKP Viability in Human Serum Following Probiotic CFS Exposure

To determine if probiotic CFS sensitizes CRKP to innate immune factors, overnight cultures of CRKP were pre-exposed to either probiotic CFS or control MRS medium (1% inoculum) for 3 h at 37°C. To enable comparison of serum-mediated killing under conditions where CFS pre-exposure alone did not substantially reduce CRKP viability, CRKP was pre-exposed to each probiotic CFS at sub-inhibitory concentrations of 1/2 MIC and 1/4 MIC, alongside an MRS broth-only control, for 3 h at 37°C. MIC-level pre-exposure was not included in this experiment, as it can itself reduce CRKP viability within the 3 h pre-exposure period, which would confound the interpretation of subsequent serum-mediated killing. After treatment, 100 μL of the bacterial culture was transferred to an equal volume of sterile human blood serum (H4522, Sigma-Aldrich, USA) in 96-well plates and incubated for 30 min at 37°C. To isolate the individual contribution of serum-mediated killing from that of CFS pre-treatment, CRKP receiving no CFS pre-exposure (MRS broth alone) was also exposed to serum under the same conditions, alongside a parallel set of MRS-only samples not exposed to serum, which served as the reference growth control. Subsequently, 10 μL from each well was streaked onto MacConkey agar for viable colony counting [27]. CRKP survival in each condition was calculated as a percentage relative to the MRS-only, serum-free control, which was set at 100%. The experiment was performed in triplicate.

### Transmission Electron Microscopy Analysis of CRKP Capsular Structure Following Probiotic CFS Treatment

Aliquots of CRKP suspension were incubated with equal volumes of probiotic CFS at 37°C for 24 h, while untreated CRKP cells incubated with fresh MRS media served as controls. After incubation, the cells were pelleted, washed twice with PBS, and immediately fixed for electron microscopy. The bacterial pellets were fixed in 2.5% glutaraldehyde (Sigma-Aldrich) prepared in 0.1 M cacodylate buffer (Thermo Fisher, USA) (pH = 7.4) overnight at 4°C. The samples were then washed in buffer, post-fixed in 1% osmium tetroxide for 1 h, then dehydrated through a graded ethanol series, followed by progressive incubation with propylene oxide and Epon 812, and finally embedded in 100% Epon 812 resin [28]. Ultrathin sections were prepared using an EM UC7 ultramicrotome (Leica, Austria) and placed on 100–150 mesh copper grids. The sections were then stained with uranyl acetate and lead citrate before imaging with a Transmission electron microscopy (TEM) (JEM-1400 Plus at 120 kV, JEOL, Japan). Digital images were captured at multiple magnifications to assess capsular thickness and structural integrity. Comparisons of capsule thickness and rupture were made between CFS-treated and untreated CRKP cells.

### Quantitative Analysis of Capsule Biosynthesis Gene Expression

The effect of probiotic strains’ CFS on the expression of the *gal*F gene, which is involved in capsule biosynthesis in CRKP, was evaluated. CRKP was cultured overnight in MacConkey broth, then adjusted to an OD_600_ = 0.1–0.2, and exposed to sub-inhibitory concentrations of CFS (¼ MIC, ½ MIC, and MIC) at 37°C for 24 h. Control cultures received MRS broth alone. Total RNA was extracted using the Qiagen RNA extraction kit (Germany), and cDNA synthesis was performed using the iScript cDNA Synthesis Kit (Bio-Rad). qPCR was conducted with SYBR Green Supermix (Bio-Rad) on a CFX96 Real-Time PCR platform (Applied Biosystems, USA). The transcript abundance of *gal*F was normalized to 16S rRNA using the ^ΔΔ^Ct method [9]. The mRNA levels of 16S rRNA from *K. pneumoniae* were used as an internal control to verify sample loading. Reactions were conducted in technical triplicate. Primer details are provided in Fig. 6E.

## Results

### Identification of Probiotics Strains

The probiotic candidate strains used in this study were obtained from the PMC probiotic bank. The purity of each isolate was assessed through bacterial 16S rRNA genome sequencing (Table 1). *L. plantarum* isolate showed 99% sequence identity with multiple reference strains in the NCBI database, including JCM 1149 and NBRC 15891. The *Bacillus* isolate exhibited 99% identity with *B. velezensis* strains (*e.g.*, FZB42). The *Lactobacillus* isolates demonstrated 99% sequence identity with several reference strains of *L. fermentum* and *L. sakei*, confirming their taxonomic assignment.

### Growth Inhibition of CRKP by Probiotic CFS

**Fig. 1:**
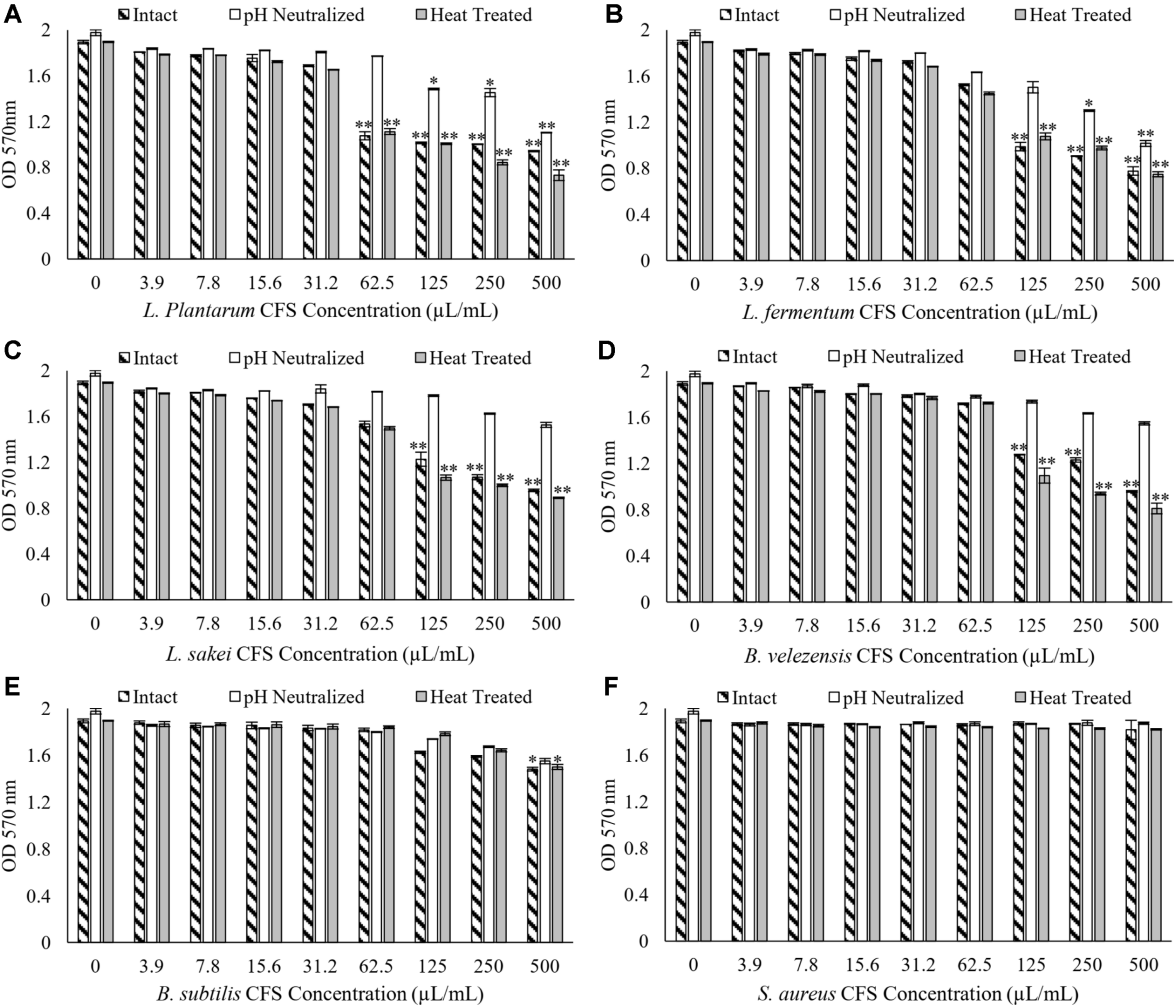
Concentration-dependent growth inhibition of CRKP by probiotic CFS, and effect of pH neutralization, heat treatment, and non-probiotic CFS. To evaluate the growth-inhibitory effects of probiotic CFS on CRKP, a concentration-dependent growth inhibition assay was performed after 24 h of co-culture. CRKP cells were exposed to two-fold serial dilutions of CFS, and growth was quantified by measuring optical density (OD) at 570 nm and compared to the untreated control group. The "0" concentration represents the growth control (fresh MRS broth alone, pH 5.5) rather than a zero-dose CFS dilution. (**A**) *L. plantarum* CFS significantly reduced CRKP growth starting at 62.5 μL/mL and showed the strongest inhibitory effect. In comparison, (**B**) *L. fermentum* and (**C**) *L. sakei* CFS exhibited similar growth-inhibition patterns, with minimum inhibitory concentrations (MICs) of 125 μL/mL. Meanwhile, (**D**) *B. velezensis* CFS also reduced CRKP growth but demonstrated relatively weaker efficacy at the same concentration, indicating strain-specific differences in antimicrobial activity. pH-neutralized and heat-treated CFS from all four probiotic strains retained significant inhibitory activity comparable to intact CFS, indicating that the observed antimicrobial effect is not solely attributable to low pH and is largely heat-stable. (**E, F**) CFS from the non-probiotic strains *B. subtilis* and *S. aureus* showed minimal or no inhibitory effect on CRKP growth across the same concentration range, supporting that the antimicrobial activity is associated with probiotic-characteristic metabolites rather than being a general feature of bacterial CFS. The antimicrobial effects are presented as mean SD. The experiment was performed in independent technical triplicates (n = 3), and statistical analysis was performed using one-way ANOVA followed by Tukey’s post hoc test (*** *p* < 0.001; ** *p* < 0.01; * *p* < 0.05).

The growth-inhibitory effects of CFS derived from candidate probiotic strains against CRKP were evaluated after 24 h of co-culture (Fig. 1). The “0” concentration group corresponds to unfermented MRS broth alone (pH 5.5), serving as the growth control rather than a CFS-free dilution point. Exposure to *L. plantarum* CFS resulted in a clear, concentration-dependent reduction in CRKP growth (Fig. 1A). No significant effects were observed at lower concentrations (1.95–31.2 μL/mL), but notable and statistically significant inhibition became evident starting at 62.5 μL/mL and intensified at higher concentrations (125–500 μL/mL), indicating strong antimicrobial activity. In contrast, *L. fermentum* CFS showed minimal effects on CRKP growth at concentrations up to 62.5 μL/mL (Fig. 1B); however, significant growth suppression was first detected at 125 μL/mL and was maintained at 250 and 500 μL/mL, establishing a MIC of 125 μL/mL. A similar inhibitory pattern was observed for *L. sakei* CFS (Fig. 1C), where CRKP growth remained comparable to the untreated control at lower concentrations but was significantly reduced at 125 μL/mL and further decreases at higher doses, also yielding an MIC of 125 μL/mL. Treatment with *B. velezensis* CFS resulted in comparatively weaker antimicrobial effects (Fig. 1D), as CRKP growth remained largely unaffected at lower concentrations, with only moderate but statistically significant inhibition observed at 125 μL/mL and above, corresponding to an MIC of 125 μL/mL. Overall, Probiotic CFS demonstrate strain-specific antimicrobial activities against CRKP, with *L. plantarum* showing the strongest inhibitory effect, followed by *L. fermentum* and *L. sakei*, while *B. velezensis* exhibited comparatively lower efficacy. To determine whether this antimicrobial activity was dependent on the acidic pH of the CFS or on heat-labile components, intact CFS was compared with pH-neutralized and heat-treated CFS across the same concentration range (Fig. 1A–1D). For all four probiotic strains, both pH-neutralized and heat-treated CFS retained significant inhibitory activity against CRKP at concentrations of 125–500 μL/mL, although the degree of inhibition by pH-neutralized CFS was modestly reduced compared with intact CFS at the lowest effective concentrations. This indicates that the antimicrobial activity of probiotic CFS is not solely attributable to its low pH and is largely retained after heat treatment. In contrast, CFS derived from the non-probiotic strains *B. subtilis* and *S. aureus* showed minimal (*B. subtilis*, significant only at 500 μL/mL) or no significant inhibitory effect on CRKP growth across the entire concentration range tested (Fig. 1E and 1F), supporting that the antimicrobial activity observed for the probiotic strains is associated with probiotic-characteristic metabolites rather than being a general feature of bacterial CFS.

### Time-Dependent Progressive Bactericidal Effects against CRKP

**Fig. 2:**
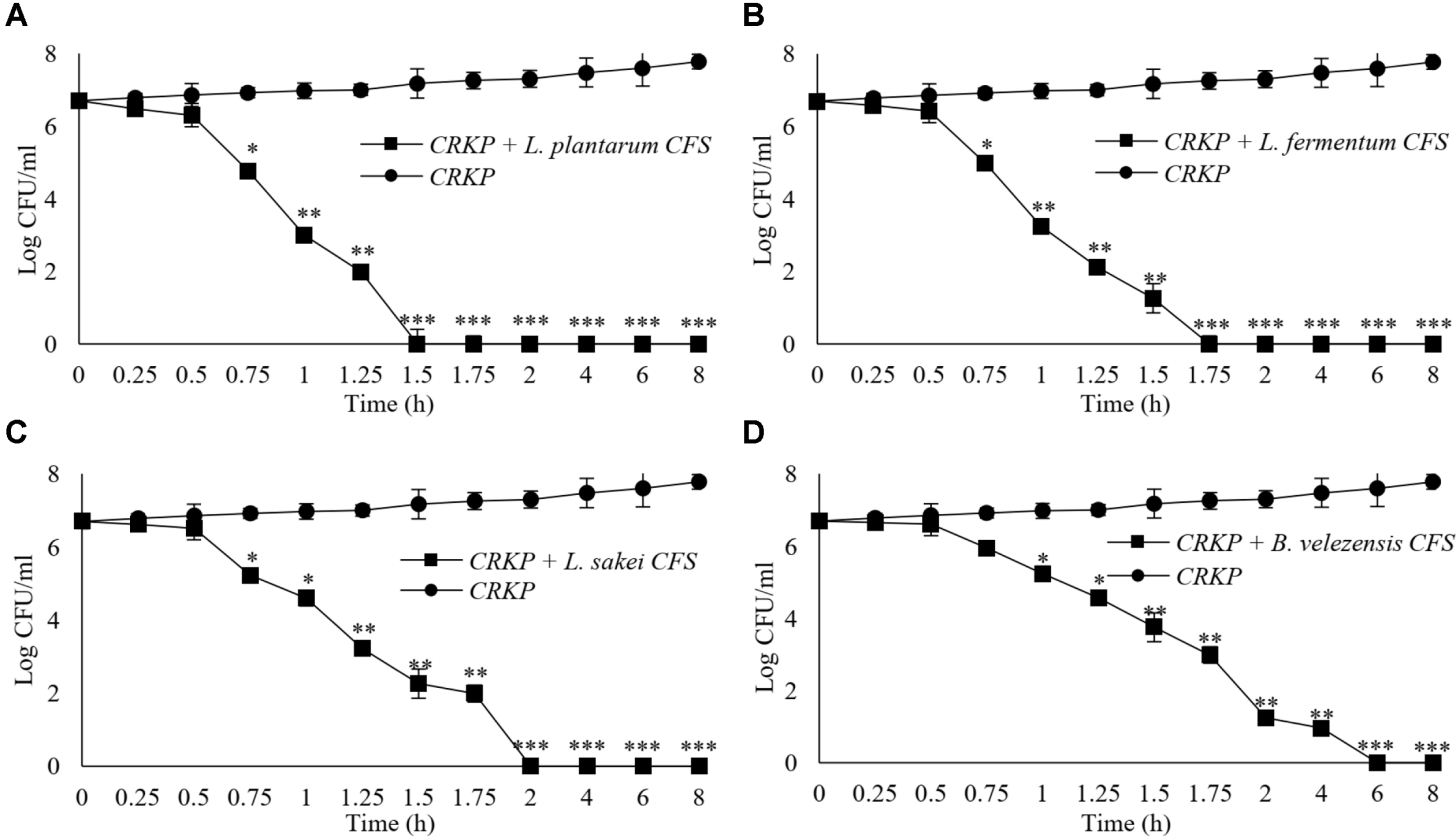
Bactericidal activity rate of probiotic candidates CFS against CRKP. To evaluate the time-dependent killing effects of CFS from selected probiotic candidates, CRKP cultures were exposed to each CFS for 8 h, and viable bacterial counts (log CFU/mL) were quantified at specific time points. (**A**) *L. plantarum* CFS induced a rapid decline in CRKP viability starting at 0.75 h, reaching complete growth suppression by 1.5–2 h. (**B**) *L. fermentum* CFS exhibited a similar bactericidal profile, with significant reductions observed after 1 h and complete killing by approximately 2 h. (**C**) *L. sakei* CFS demonstrated progressive killing activity, achieving near-complete elimination by 2–4 h, and (**D**) *B. velezensis* CFS showed a comparatively slower but sustained bactericidal effect, with marked reduction in viable counts observed after 2 h and complete suppression by 4–6 h. All probiotic CFS samples induced a progressive and significant reduction in CRKP viability compared to the untreated control at corresponding time points. Statistical significance was determined by one-way ANOVA followed by Tukey’s post hoc test (*** *p* < 0.001; ** *p* < 0.01; * *p* < 0.05).

The time-dependent bactericidal activity of CFS derived from selected probiotic candidates against CRKP was assessed by monitoring viable bacterial counts (log CFU/mL) over an 8 h incubation period (Fig. 2). In the untreated control groups, CRKP showed stable or slightly increased viable counts throughout the experiment, indicating active bacterial growth in the absence of CFS. In contrast, treatment with *L. plantarum* CFS (Fig. 2A) caused a rapid and significant reduction in CRKP viability, with a notable decline observed within 1 h, followed by near-complete bacterial killing by 2 h, which was maintained through 8 h. Similarly, exposure to *L. fermentum* CFS (Fig. 2B) resulted in a progressive decrease in viable CRKP counts, with significant bactericidal effects apparent from 1–1.25 h, and complete or near-complete elimination occurring by approximately 2 h. Treatment with *L. sakei* CFS (Fig. 2C) also demonstrated a strong time-dependent killing effect, beginning with a gradual reduction in CFU counts within the first hour and reaching undetectable levels by 2 h, which persisted for the remainder of the experiment. In comparison, *B. velezensis* CFS (Fig. 2D) showed slower bactericidal kinetics, exhibiting a more gradual decline in CRKP viability. However, a significant reduction in CFU counts was still observed over time, leading to substantial bacterial killing by 4 to 6 h and sustained suppression up to 8 h. Overall, all tested probiotic CFS exhibited bactericidal activity against CRKP, with *L. plantarum*, *L. fermentum*, and *L. sakei* demonstrating rapid and potent killing effects, while *B. velezensis* displayed effective but comparatively delayed bactericidal activity.

### Safety Profiles of Probiotic Candidates

**Fig. 3:**
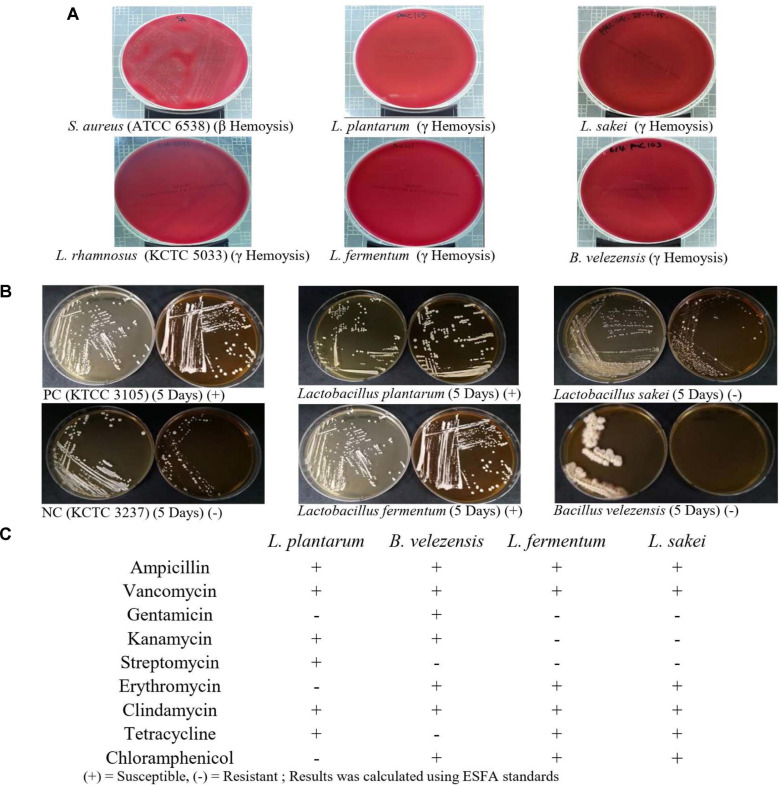
Safety and functional characterization of selected probiotic strains. To assess the safety and functional potential of the candidate probiotics, hemolytic activity, bile salt deconjugation, and antibiotic susceptibility were assessed using standard phenotypic assays. (**A**) None of the tested probiotic strains exhibits hemolytic activity on blood agar plates, in contrast to the clear hemolysis zone produced by the positive control, *S. aureus* ATCC 6538. (**B**) Bile salt deconjugation ability is evaluated on agar plates with (right side) and without (left side) taurodeoxycholic acid (TDCA), using *L. rhamnosus* KCTC 5033 and *L. plantarum* KCTC 3105 as negative and positive controls, respectively. Under these conditions, all candidate strains show bile salt tolerance, except for *Bacillus velezensis*, which shows sensitivity. (**C**) Antibiotic susceptibility and resistance profiles for the selected strains are determined against nine different antibiotics. Collectively, these results confirm the safety of the candidates with respect to hemolytic activity while highlighting variable bile salt tolerance and distinct antibiotic resistance patterns.

The safety and functional properties of the selected probiotic candidates were assessed using hemolysis, bile salt deconjugation, and antibiotic susceptibility assays (Fig. 3).

As shown in Fig. 3A, none of the tested probiotic strains exhibited hemolytic activity on blood agar plates. In contrast, *S. aureus* ATCC 6538 displayed clear β-hemolysis, confirming the assay's validity, while *L. rhamnosus* KCTC 5033 served as a non-hemolytic negative control. Importantly, none of the tested probiotic strains, including *L. plantarum*, *L. fermentum*, *L. sakei*, and *B. velezensis*, demonstrated any hemolytic activity, indicating a favorable safety profile.

Fig. 3B presents the bile salt deconjugation and tolerance profiles of the candidate strains in TDCA-supplemented media. *L. plantarum* KCTC 3105 was included as a positive control, while *L. rhamnosus* KCTC 5033 served as a negative control. Under these conditions, *L. plantarum* and *L. fermentum* showed robust growth in the presence of TDCA, indicating bile salt tolerance. In contrast *L. sakei* showed limited growth and *B. velezensis* demonstrated significant sensitivity, suggesting strain-specific variations in bile salt resistance.

As presented in Fig. 3C, the candidate strains displayed distinct antibiotic susceptibility and resistance profiles. Although all strains were generally susceptible to clinically relevant antibiotics like ampicillin and vancomycin, variability was noted in their responses to aminoglycosides, macrolides, and tetracycline-class antibiotics, indicating intrinsic and strain-dependent resistance.

### Modulation of Capsular Polysaccharide Synthesis and Serum Susceptibility of CRKP by Probiotics CFS

**Fig. 4:**
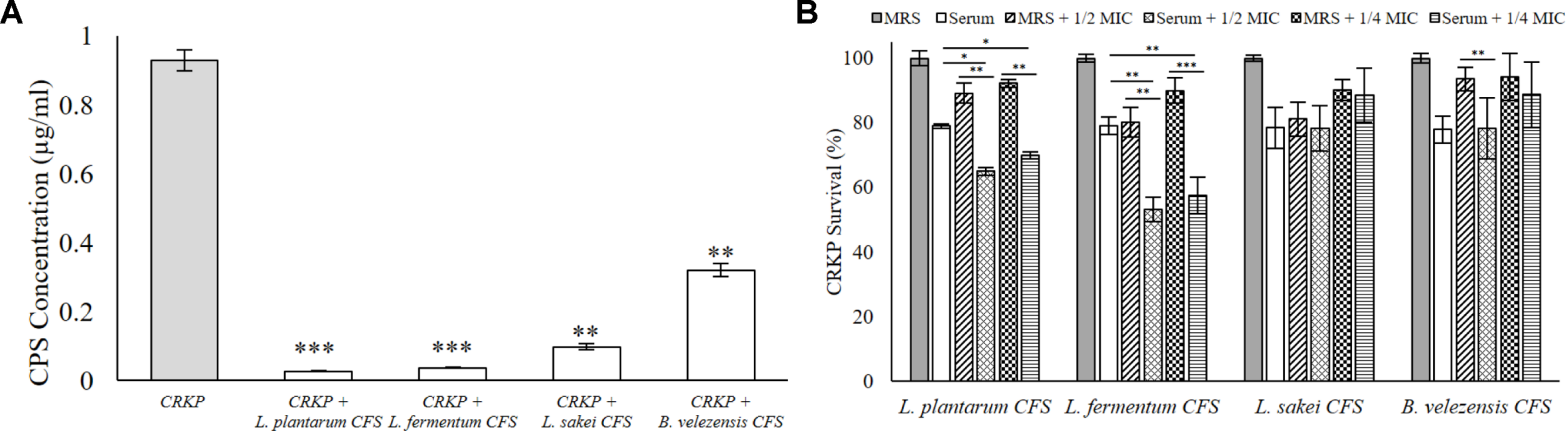
Effects of probiotic candidate CFS on CRKP capsular polysaccharide production and serum killing susceptibility. To investigate how probiotic CFS modulate the virulence attributes of CRKP, capsular polysaccharide (CPS) concentrations and serum survival rates were quantified following co-incubation. Results illustrate how probiotic CFS modulate CRKP virulence attributes. CRKP cultures were incubated with each probiotic CFS, after which CPS concentration and serum survival were quantified using a calorimetric Phenol-sulfuric acid assay and human blood serum. (**A**) Co-incubation with probiotic CFS significantly reduced CPS production compared to CRKP co-cultured in fresh MRS broth. (**B**) CRKP was pre-exposed to each probiotic CFS at sub-inhibitory concentrations of 1/2 MIC and 1/4 MIC, or to MRS broth alone, for 3 h prior to exposure to human serum or MRS broth alone. CRKP survival in each condition is expressed relative to the MRS-only, serum-free control (set as 100%). Serum exposure alone modestly reduced CRKP survival relative to the MRS-only control, reflecting partial innate serum resistance; this serum-only condition was assessed once and used as a common reference across all four strain panels. Pre-exposure to sub-inhibitory CFS alone did not substantially reduce CRKP viability for any strain. For *L. plantarum* and *L. fermentum*, pre-exposure to sub-inhibitory CFS significantly reduced CRKP survival in serum compared with serum alone, indicating enhanced serum sensitization. In contrast, no significant reduction in serum survival relative to serum alone was observed for *L. sakei* or *B. velezensis* at either sub-inhibitory concentration. Statistical significance was determined using oneway ANOVA with Tukey’s post hoc comparisons (*** *p* < 0.001; ** *p* < 0.01; * *p* < 0.05).

The effects of probiotic CFS on key virulence traits of CRKP were evaluated by measuring CPS production and susceptibility to human serum–mediated killing (Fig. 4). Treatment with probiotic candidates’ CFS significantly reduced CPS production in CRKP (Fig. 4A). Untreated CRKP produced high levels of CPS, reflecting a strong encapsulation phenotype. In contrast, co-incubation with CFS from all tested probiotic candidates led to a significant decrease in CPS concentration, suggesting suppression of capsule biosynthesis. Among the strains tested, *L. plantarum*, *L. fermentum*, and *L. sakei* CFS caused a marked reduction in CPS levels compared to the untreated control, while *B. velezensis* CFS also reduced CPS production, albeit to a lesser extent. To determine whether probiotic CFS enhances CRKP susceptibility to serum-mediated killing beyond the effect of serum alone, CRKP was pre-exposed to sub-inhibitory concentrations of CFS (1/2 MIC and 1/4 MIC) or MRS broth, followed by exposure to either human serum or MRS broth alone (Fig. 4B). Serum exposure alone reduced CRKP survival to approximately 79% relative to the MRS-only control, reflecting the partial innate serum resistance conferred by the CRKP capsule; this serum-only condition was assessed once and used as a common reference across all four strain panels in Fig. 4B. Pre-exposure to sub-inhibitory CFS alone (without serum) did not substantially reduce CRKP viability for any strain, confirming that CRKP remained largely viable at the time of serum challenge under all conditions tested. For *L. plantarum* CFS, survival in serum was further reduced from 79.0 0.7% (serum alone) to 65.3 1.3% and 70.9 1.1% following pre-exposure to 1/2 MIC and 1/4 MIC CFS, respectively (*p* < 0.05 for both vs. serum alone), indicating a significant additional reduction in serum survival attributable to CFS pre-treatment. A comparable pattern was observed for *L. fermentum* CFS, where serum survival decreased from 79.0 2.7% (serum alone) to 53.3 3.7% at 1/2 MIC and 57.6 5.6% at 1/4 MIC (*p* < 0.01 for both comparisons vs. serum alone), representing the most pronounced sensitization among the four strains. In contrast, CRKP pre-exposed to *L. sakei* or *B. velezensis* CFS showed serum survival comparable to the serum-alone control at both sub-inhibitory concentrations (78.3 7.0% and 88.6 8.4% for *L. sakei*; 78.3 9.5% and 88.7 10.0% for *B. velezensis*, at 1/2 MIC and 1/4 MIC, respectively), with no significant difference from serum alone, indicating no additional sensitization to serum killing for these two strains at the concentrations tested. These findings indicate that sub-inhibitory concentrations of *L. plantarum* and *L. fermentum* CFS enhance CRKP susceptibility to complement-mediated serum killing beyond the effect of serum alone, despite these concentrations having minimal direct effect on CRKP viability. This sensitizing effect was not observed for *L. sakei* or *B. velezensis* CFS at the sub-inhibitory concentrations tested.

### Structural Changes in the Capsule of CRKP Induced by Probiotics CFS

**Fig. 5:**
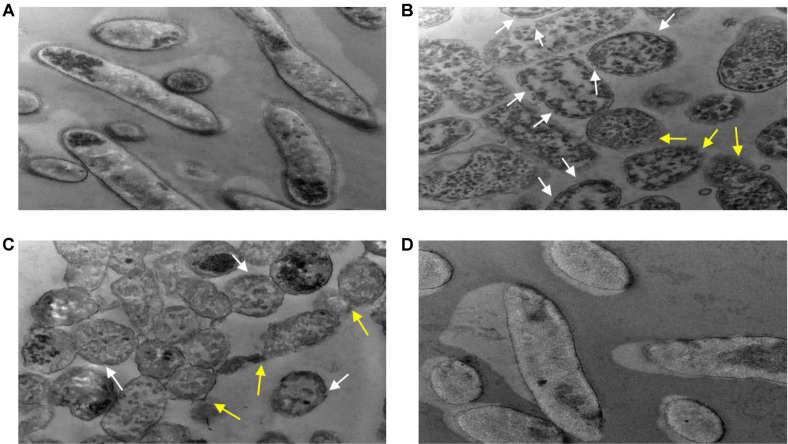
TEM analysis of CRKP capsule disruption by probiotic CFS. To analyze structural alterations in the CRKP capsule following exposure to different probiotic CFS, representative TEM micrographs were obtained to visualize the pathogen’s capsule structure. (**A**) Untreated control cells exhibit intact morphology, characterized by a continuous, uniformly thick, and electron-dense capsular layer surrounding the bacterial cell envelope, with no evidence of structural discontinuity or cytoplasmic leakage. (**B**) In contrast, exposure to *L. plantarum* CFS resulted in significant disruption of the capsular architecture, including multiple pore-like defects and discontinuities within the capsule matrix (white arrows), frequently accompanied by the extrusion of cytoplasmic contents into the extracellular space (yellow arrows), indicating severe membrane destabilization. (**C**) Similarly, treatment with *L. fermentum* CFS induced extensive disorganization of the capsule, irregular thinning, and pronounced perforation of the capsular layer (white arrows), along with significant cytoplasmic leakage (yellow arrows) suggesting compromised cell envelope integrity and progressive structural collapse. (**D**) In comparison, cells treated with *B. velezensis* CFS showed a noticeable reduction in capsule thickness and electron density relative to untreated controls; however, distinct pore formation or overt cytoplasmic leakage was not readily apparent, indicating partial capsule attenuation without extensive perforation. Yellow arrows indicate sites of cytoplasmic leakage, while white arrows highlight capsule pores.

TEM analysis revealed distinct ultrastructural alterations in the capsular architecture of CRKP after exposure to probiotic CFS (Fig. 5). As illustrated in Fig. 5A, untreated CRKP cells displayed a well-defined, continuous capsule layer that appeared thick and uniformly enveloping the bacterial cell, providing a consistent protective barrier with no visible discontinuities, perforations, or cytoplasmic leakage. In contrast, CRKP cells treated with CFS derived from different probiotic strains showed significant alterations in capsule morphology. TEM micrographs demonstrated that treatment with *L. plantarum* CFS resulted in multiple pore-like defects within the capsular matrix, leading to clear discontinuities in the previously continuous capsule layer (Fig. 5B). These structural breaches were often linked to the extrusion of intracellular contents into the extracellular space, suggesting a loss of membrane integrity and severe compromise of the bacterial envelope. Similar effects were observed with *L. fermentum* CFS (Fig. 5C), where widespread pore formation was frequently associated with visible cytoplasmic leakage, indicating a progressive structural collapse of both the capsule and the underlying cell envelope. In contrast, CRKP cells exposed to *B. velezensis* CFS displayed a different pattern of alteration (Fig. 5D). Although the capsular layer remained continuous, with no evident pore formation or cytoplasmic leakage, the capsule thickness was significantly reduced compared to untreated controls. The capsular layer appeared thinner and less electron-dense, indicating partial degradation without overt structural perforation. Collectively, these findings demonstrate strain-dependent differences in capsular disruption: *L. plantarum* and *L. fermentum* CFS cause severe perforation-associated damage, while *B. velezensis* CFS primarily led to capsule thinning without extensive envelope rupture.

### Downregulation of CRKP Capsular Gene *gal*F by Probiotic CFS

**Fig. 6:**
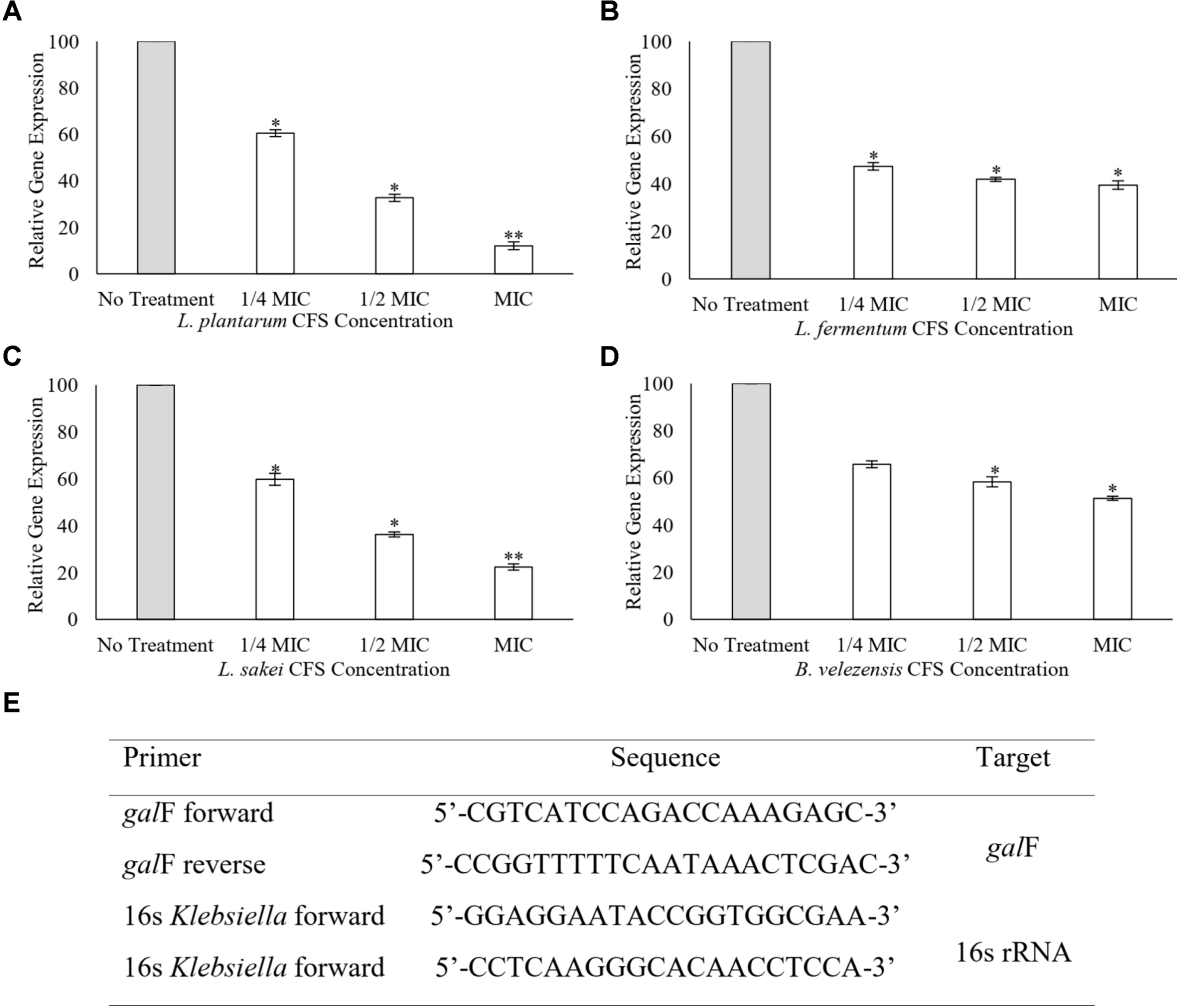
Probiotic CFS treatment reduced the relative expression of the *gal*F gene in CRKP. To determine the impact of probiotic CFS on the molecular mechanisms of capsule biosynthesis, the relative expression of the *gal*F gene was quantified via qRT-PCR following a 3 h exposure to MIC and sub-MIC CFS concentrations. (**A**) *L. plantarum* CFS significantly reduced *gal*F expression in a concentration-dependent manner. (**B**) *L. fermentum* CFS also suppressed *gal*F expression, with the strongest effect observed at MIC. (**C**) *L. sakei* CFS produced effects similar to *L. plantarum*, showing marked downregulation of *gal*F. (**D**) *B. velezensis* CFS caused weaker suppression of *gal*F compared to the lactobacilli strains. Probiotic CFS can downregulate capsule biosynthesis genes in CRKP, with strain-specific differences in potency. (**E**) Primer sequences used for amplification of *gal*F and the housekeeping gene. Data are presented as mean SD of the percentile ratio relative to the untreated control group. Statistical analysis was performed using one-way ANOVA followed by Tukey’s post hoc test (*** *p* < 0.001; ** *p* < 0.01; * *p* < 0.05).

To assess the genomic interference of probiotic CFS on capsular formation in CRKP, relative *gal*F gene expression levels were measured following 3 h exposure to sub-MIC and MIC concentrations (Fig. 6). The analysis revealed that CFS significantly reduced the expression of the *gal*F gene in CRKP. Relative to the untreated control, *L. plantarum* CFS induced a clear concentration-dependent downregulation of *gal*F, with progressively greater suppression observed at 1/4 MIC, 1/2 MIC, and MIC (Fig. 6A). Treatment with *L. fermentum* CFS also significantly decreased *gal*F expression at all tested concentrations, with the strongest inhibition detected at MIC (Fig. 6B). A similar pattern of marked *gal*F downregulation was observed following exposure to *L. sakei* CFS (Fig. 6C). In contrast, *B. velezensis* CFS resulted in a weaker yet statistically significant reduction in *gal*F expression compared to the lactobacilli-derived CFS (Fig. 6D). Overall, all probiotic CFS treatments significantly suppressed *gal*F expression in CRKP compared to the untreated control, with clear strain-specific differences in inhibitory potency.

## Discussion

CRE, particularly CRKP, are among the most pressing global threats of antimicrobial resistance due to their high mortality rates, rapid spread, and limited treatment options [29]. The traditional development of antibiotics has not kept pace with the rise of resistance, necessitating the need for alternative therapeutic approaches [30, 31]. Microbiome-based therapeutics have emerged as a promising approach, with mechanisms of action that differ significantly from conventional bactericidal or bacteriostatic antibiotics [31]. In this context, the present study evaluates probiotic-derived candidate strains against CRKP, focusing on their efficacy, safety, and underlying mechanisms of action.

In our previous studies, we systematically evaluated probiotic-derived strategies targeting CRKP, establishing a strong experimental and conceptual foundation for the current work. Across four independent investigations, we consistently demonstrated that *L. plantarum*, *L. fermentum*, *L. sakei*, and *B. velezensis* exhibit significant anti-CRKP activity. These were supported by reproducible assays, standardized antimicrobial testing protocols, and mechanistic validation [7, 8, 10, 14]. Importantly, these studies confirmed the safety and efficacy of the live strains, the present work focuses on Cell-Free Supernatants (CFS) to explore a mechanistically defined and clinically feasible antimicrobial modality that avoids the risks associated with live bacterial administration in immunocompromised patients [32]. This accumulated evidence warranted a focused evaluation of the metabolites within the CFS as the primary therapeutic agents. Consequently, this current study was conducted exclusively using CFS to investigate its specific inhibitory and anti-virulence effects [33].

These findings indicate that probiotic-derived metabolites can be effective against CRKP. The CFS from all four probiotic candidates significantly inhibited CRKP growth in a dose-dependent manner, with notable strain-specific differences in potency. *L. plantarum* CFS exhibited the strongest inhibitory effect, leading to substantial CRKP growth suppression. Importantly, this strain also demonstrated a favorable safety profile, including the absence of hemolytic activity, appropriate bile salt deconjugation, and preserved antibiotic susceptibility. These findings further support the importance of comprehensive safety screening as a critical component of microbiome therapeutic development pipelines [34]. While *L. fermentum* and *L. sakei* displayed comparable inhibitory profiles, in addition to their promising safety aspects, this contributes to their overall biosafety and compatibility with host tissues. Although *B. velezensis* CFS also reduced CRKP growth, its comparatively weaker efficacy highlights the variability in antimicrobial metabolite production among probiotic strains. Moreover, its excessive bile salt deconjugation activity may represent an undesirable metabolic or ecological characteristic, particularly in the context of gut-delivered therapeutics [35]. These results emphasize the importance of strain-level selection when developing microbiome-derived therapeutics and suggest that distinct metabolite profiles play different roles in pathogen suppression [36, 37]. Beyond growth inhibition, the study offers crucial evidence that probiotic CFS exhibit genuine bactericidal activity against CRKP. Time-kill assays showed a significant and progressive decrease in viable CRKP counts following exposure to each CFS. This confirms that these metabolites not only impose bacteriostatic pressure but also actively reduce bacterial viability. This distinction is especially important in the context of severe CRE infections, where bactericidal activity is often necessary for achieving effective clinical outcomes [38-41]. The observed time-dependent killing suggests sustained antimicrobial action, which may be beneficial for preventing regrowth and the emergence of resistance [42].

To determine whether the antimicrobial activity of probiotic CFS was driven by low pH rather than specific metabolites, intact CFS was compared with pH-neutralized and heat-treated preparations. Inhibitory activity against CRKP was still observed after pH neutralization and heat treatment, suggesting that acidity may not be the sole factor underlying the antimicrobial effect, and that at least part of the active components may not be heat-labile [43, 44]. To further confirm that this activity reflects probiotic-specific metabolites rather than a general feature of bacterial CFS, supernatants from the non-probiotic strains *B. subtilis* and *S. aureus* were tested under identical conditions and showed minimal or no inhibitory effect against CRKP, despite comparable preparation procedures.

The CPS, lipopolysaccharide (LPS), siderophore-mediated iron-acquisition systems, and various antibiotic resistance genes are key players in the pathogenesis and persistence of CRKP [45]. Specifically, CPS is a key virulence factor that protects the bacterium against host immune surveillance by preventing the activation of complement, phagocytosis, and serum-mediated bactericidal activity-the major elements of the innate immune response [27]. The mechanistic discovery of this study reveals that the probiotic candidate-derived CFS inhibits CPS production in CRKP, resulting in a truncated, less dense capsular architecture. This structural attenuation increases the exposure of the underlying cytoplasmic membrane, thereby facilitating the deposition of serum complement components and the subsequent formation of lethal membrane attack complexes. By doing so, the CFS considerably diminished an important determinant of immune evasion, thus sensitizing CRKP to clearance by host immunity [46, 47]. This anti-virulence strategy symbolizes a conceptual shift in therapeutic perspective, in which, instead of direct killing of bacteria, functional disarmament is employed, potentially imposing less selective pressure on resistance evolution [47].

TEM analysis provided direct ultrastructural evidence that probiotic-derived CFS disrupts capsular integrity in CRKP. Untreated CRKP cells displayed a dense, continuous, and well-organized polysaccharide capsule surrounding the bacterial envelope, consistent with the hypercapsulated phenotype associated with increased resistance to host defenses and antimicrobial agents [27]. Exposure to *L. plantarum* and *L. fermentum* CFS resulted in significant structural damage, including disrupted capsule architecture, pore formation, and cytoplasmic leakage. These observations indicate that probiotic-derived metabolites not only interfere with CPS production but also compromise bacterial envelope integrity, ultimately leading to leakage of intracellular contents and cell death [48, 49]. These ultrastructural changes align with the strong bactericidal activity observed in previous growth inhibition and time-kill assays, indicating that capsule disruption is a critical mechanistic step in the killing of CRKP by probiotic-derived metabolites. Treatment with *B. velezensis* CFS resulted in a distinct, yet comparatively milder phenotype, characterized by a noticeable reduction in capsule thickness without apparent pore formation or cytoplasmic leakage. This finding suggests that while *B. velezensis* metabolites can interfere with capsule biosynthesis or maintenance, they may be less effective in directly causing membrane or capsule rupture [49]. These differential ultrastructural outcomes correspond with the relatively weaker antimicrobial efficacy of *B. velezensis* CFS observed in growth inhibition assays, further emphasizing the strain-specific nature of probiotic metabolite activity [50].

These ultrastructural findings were strongly supported at the molecular level by the significant downregulation of *gal*F, a key gene involved in CPS biosynthesis which is highly conserved through various *K. pneumoniae* serotypes [51]. Reduced *gal*F expression provides a plausible mechanistic explanation for the observed decline in capsule production and the altered capsular morphology visualized by TEM [52]. Specifically, CPS reduction unmasks surface lipopolysaccharide and outer membrane proteins, enabling C3b deposition via the classical and alternative complement pathways. This initiates terminal complement cascade assembly, culminating in C5b-9 membrane attack complex formation, transmembrane pore insertion, and ultimately bacterial lysis consistent with the increased serum susceptibility observed in CFS-treated CRKP [53, 54]. This suggests that microbiome-derived therapeutics can interact with the innate immune response and likely re-establish host-mediated clearance of bacteria, even against highly drug-resistant pathogens. Importantly, strains that produced the greatest reduction in *gal*F expression and CPS abundance also exhibited the strongest antibacterial and serum-sensitizing effects, suggesting that capsule suppression is not merely an associated phenotype but a major contributor to the antimicrobial activity of probiotic-derived metabolites against CRKP [55, 56]. A further consideration is whether the enhanced serum susceptibility of CRKP following CFS pre-exposure was simply a consequence of the bactericidal activity of the CFS itself. To address this, we compared CRKP survival in serum alone with survival in serum following pre-exposure to sub-inhibitory CFS concentrations (1/2 MIC and 1/4 MIC), conditions under which CFS alone did not substantially reduce CRKP viability. For *L. plantarum* and *L. fermentum* CFS, pre-exposure significantly reduced serum survival relative to serum alone, indicating that these two strains sensitize CRKP to serum killing through a mechanism beyond their own modest direct effect on viability, plausibly linked to the concurrent suppression of capsular polysaccharide synthesis. In contrast, *L. sakei* and *B. velezensis* CFS did not significantly alter CRKP survival relative to serum alone at either sub-inhibitory concentration, suggesting that serum sensitization by these two strains, where observed at higher CFS concentrations, may depend more directly on their own antimicrobial activity rather than a distinct anti-virulence mechanism. [57].

These findings may have implications beyond CRE infection, as capsule suppression represents a broadly relevant anti-virulence mechanism that could be applied against a wide range of encapsulated pathogens, including other members of the *Enterobacteriaceae* family [49]. In addition, microbiome-derived metabolite therapies are consistent with new therapeutic strategies that seek to modulate pathogen behavior rather than indiscriminate microbial killing [37]. This strategy could be beneficial for preserving commensal microbial communities while selectively abolishing pathogenic traits, which is significant for long-term patient outcomes. In the present study, the combination of direct antibacterial activity, capsule suppression, immune sensitization, and favorable safety characteristics suggests that probiotic-derived CFS may represent a promising therapeutic platform against CRKP. Importantly, the observed relationship between CPS inhibition, *gal*F downregulation, serum sensitization, and bacterial killing supports capsule suppression as a central therapeutic axis rather than merely an associated phenotype. Despite the promising antibacterial and anti-virulence activity demonstrated by probiotic-derived CFS against CRKP in the present study, several limitations should be acknowledged. First, the experiments were performed using a single CRKP strain, and validation across multiple clinical isolates and capsular serotypes will be necessary to confirm the broader applicability of these findings. In addition, although the observed activity is likely mediated by a combination of organic acids, bacteriocin-like compounds, hydrogen peroxide, and other probiotic-derived metabolites, the relative contribution of individual components remains incompletely characterized. While the present study indicates that the antimicrobial activity is not solely dependent on the acidic pH of the CFS and persists after heat treatment, the precise identity and relative contribution of individual bioactive metabolites remain to be determined. Future studies involving metabolomic profiling, and bioactive fraction characterization will therefore be important to further define the underlying mechanisms. Furthermore, extended time-kill analyses, additional reference gene validation for qPCR normalization, and mechanistic studies incorporating alternative serum exposure models may further strengthen interpretation of the observed antibacterial effects. Finally, important translational considerations, including pharmacokinetic evaluations, administration route optimization, potential microbiome disruption, and validation in dedicated *in vivo* infection models, remain to be addressed before clinical application can be considered, particularly in immunocompromised patient populations with impaired innate immune responses.

## Conclusion

**Fig. 7:**
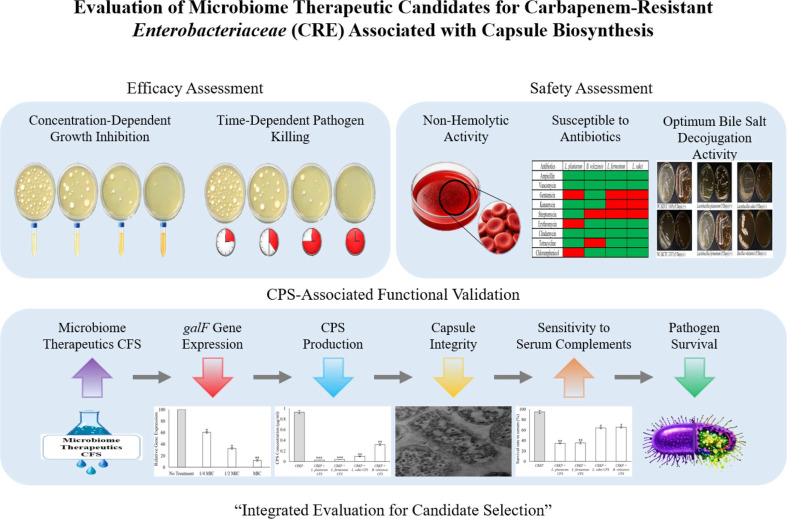
Schematic summary of microbiome therapeutic candidate evaluation targeting CPS biosynthesis in CRKP. Probiotic-derived cell-free supernatants (CFS) were evaluated for safety, antimicrobial activity, and mechanism of action against CRKP. CFS exhibited concentration-dependent growth inhibition and bactericidal activity. Mechanistically, CFS suppressed *galF* expression, leading to reduced capsular polysaccharide (CPS) production and compromised capsule integrity. This led to increased susceptibility to serum-mediated killing. CPS suppression correlated with antibacterial efficacy, enabling prioritization of candidate strains. These findings support a capsule-targeting microbiome therapeutic strategy for CRE.

The comparative evaluation of four microbiome-based therapeutic candidates against carbapenem-resistant *K. pneumoniae* revealed that probiotic-derived CFS suppressed capsular polysaccharide biosynthesis by downregulating *gal*F, sensitizing CRKP to complement-mediated killing, and establishing capsule suppression as a central and actionable therapeutic axis. Figure 7 summarizes the key findings across three dimensions: antimicrobial efficacy, safety profiles, and the underlying mechanism of action whereby suppression of capsular polysaccharide biosynthesis enhances CRKP susceptibility to host immune clearance. These results underscore the potential of microbiome-based interventions as viable alternatives to combat multidrug-resistant pathogens and provide a solid rationale for prioritizing *L. plantarum* and *L. fermentum*, which demonstrated the strongest bactericidal activity, most pronounced capsule attenuation, and most favorable safety profiles, for further preclinical validation and eventual clinical application. Future studies focusing on *in vivo* efficacy, optimal formulation, and long-term safety will be crucial for advancing these candidates toward therapeutic use.

## Figures and Tables

**Table 1:** Identification of selected probiotic strains based on 16S rRNA gene sequence analysis.

Strain	NCBI Ref.	Organism	Length	Score	Identities
*L. plantarum*	NR_115605.1	*Lactiplantibacillus plantarum* strain JCM 1149	1519	2737 bits (1482)	1483/1484 (99%)
	NR_113338.1	*Lactiplantibacillus plantarum* strain NBRC 15891	1492	2737 bits (1482)	1483/1484 (99%)
	NR_104573.1	*Lactiplantibacillus plantarum* strain CIP 103151	1527	2723 bits (1474)	1476/1477 (99%)
	NR_042394.1	*Lactiplantibacillus plantarum* strain NRRL B-14768	1474	2712 bits (1468)	1473/1475 (99%)
	NR_025447.1	*Lactiplantibacillus paraplantarum* strain DSM 10667	1502	2723 bits (1474)	1482/1486 (99%)
*B. velezencis*	NR_075005.2	*Bacillus velezensis* strain FZB42	1550	2590 bits (1402)	1406/1408 (99%)
	NR_116240.1	*Bacillus velezensis* strain CBMB205	1445	2577 bits (1395)	1404/1408 (99%)
	NR_041455.1	*Bacillus amyloliquefaciens* strain NBRC 15535	1472	2579 bits (1396)	1404/1408 (99%)
	NR_102783.2	*Bacillus subtilis* subsp. *subtilis* strain 168	1550	2573 bits (1393)	1403/1408 (99%)
	NR_024696.1	*Bacillus vallismortis* strain DSM 11031	1530	2567 bits (1390)	1402/1408 (99%)
*L. fermentum*	NR_113335.1	*Lactobacillus fermentum* strain NBRC 15885	1501	2752 bits (1490)	1495/1498 (99%)
	NR_104927.1	*Lactobacillus fermentum* strain CIP 102980	1502	2743 bits (1485)	1487/1488 (99%)
	NR_118978.1	*Lactobacillus fermentum* strain NCDO 1750	1381	2285 bits (1237)	1320/1382 (96%)
	NR_029084.1	*Lactobacillus gastricus* strain Kx156A7	1550	2410 bits (1305)	1436/1500 (96%)
	NR_028810.1	*Lactobacillus ingluviei* strain KR3	1506	2396 bits (1297)	1434/1500(96%)
*L. sakei*	NR_042443.1	*Lactobacillus sakei* subsp. *sakei* strain DSM 20017	1561	2778 bits (1504)	1508/1510 (99%)
	NR_113821.1	*Lactobacillus sakei* strain NBRC 15893	1499	2763 bits (1496)	1498/1499 (99%)
	NR_104208.1	*Lactobacillus sakei* subsp. *carnosus* strain CCUG 31331	1414	2571 bits (1392)	1407/1414 (99%)
	NR_115172.1	*Lactobacillus sakei* subsp. *sakei* strain DSM 20017	1406	2590 bits (1402)	1405/1406 (99%)
	NR_042438.1	*Lactobacillus graminis* strain G90	1548	2712 bits (1468)	1497/1511 (99%)
